# Shoot apical meristem morphoanatomy of the Mexican cactus *Mammillaria san-angelensis* and its significance for evolutionary developmental biology

**DOI:** 10.1093/aob/mcag130

**Published:** 2026-06-01

**Authors:** Brenda Anabel López-Ruiz, Valeria Romero-Gómez, Ivonne S Altamirano-Hernández, Cristian Genaro Ramírez-Castro, Delil A Chincoya, Eduardo Torres-Flores, Jerónimo Reyes-Santiago, Ulises Rosas

**Affiliations:** Jardín Botánico, Instituto de Biología, Universidad Nacional Autónoma de Mexico, Mexico City 04510, Mexico; Jardín Botánico, Instituto de Biología, Universidad Nacional Autónoma de Mexico, Mexico City 04510, Mexico; Jardín Botánico, Instituto de Biología, Universidad Nacional Autónoma de Mexico, Mexico City 04510, Mexico; Jardín Botánico, Instituto de Biología, Universidad Nacional Autónoma de Mexico, Mexico City 04510, Mexico; Posgrado en Ciencias Biológicas, Universidad Nacional Autónoma de Mexico, Mexico City 04510, Mexico; Jardín Botánico, Instituto de Biología, Universidad Nacional Autónoma de Mexico, Mexico City 04510, Mexico; Jardín Botánico, Instituto de Biología, Universidad Nacional Autónoma de Mexico, Mexico City 04510, Mexico; Posgrado en Ciencias Biológicas, Universidad Nacional Autónoma de Mexico, Mexico City 04510, Mexico; Jardín Botánico, Instituto de Biología, Universidad Nacional Autónoma de Mexico, Mexico City 04510, Mexico; Jardín Botánico, Instituto de Biología, Universidad Nacional Autónoma de Mexico, Mexico City 04510, Mexico

**Keywords:** Areole, *Arabidopsis thaliana*, Cactaceae, evolution, floral meristem, shoot apical meristem, gigantism

## Abstract

**Background and Aims:**

The shoot apical meristem (SAM) plays a key role in developmental biology by generating all aerial organs. Most research has focused on model plants, such as *Arabidopsis thaliana*, but studying plants in arid environments, such as cacti, can reveal evolutionary insights. Cacti have large SAMs that are correlated with stem size. This study examines the development of the SAM in *Mammillaria san-angelensis* and explores the relationship between SAM size and plant allometry during development.

**Methods:**

We analysed plant morphology, specifically hypocotyl and stem dimensions, along with five SAM anatomical traits across eight developmental stages, from 1 day post-germination to a 4-year-old plant, using conventional microscopy and anatomical techniques. We compared the vegetative SAM, floral meristem and areole meristem and performed correlation analysis to link SAM with plant traits.

**Key Results:**

We identified key developmental processes during the early stages of plantlet growth, including morphogenesis of the SAM and the initiation of dimorphic areole formation. We noted the increase in size and the change from two to three layers in the SAM, along with alterations in its dome formation. We found that the size of the SAM can explain the allometric growth in stem width and length.

**Conclusions:**

The correlation of larger SAM with larger stem seen in adult cacti across the Cactaceae family was recapitulated during the development of a single species (*M. san-angelensis*). Altogether, we set the developmental framework for future genetic research to characterize the evolutionary bases of SAM size increase, the meristem variants of *M. san-angelensis* and the potential of their understanding to explain the origins of gigantism, succulence and the diversity of shapes and sizes in the cacti family.

## Graphical Abstract

**Graphical Abstract:**
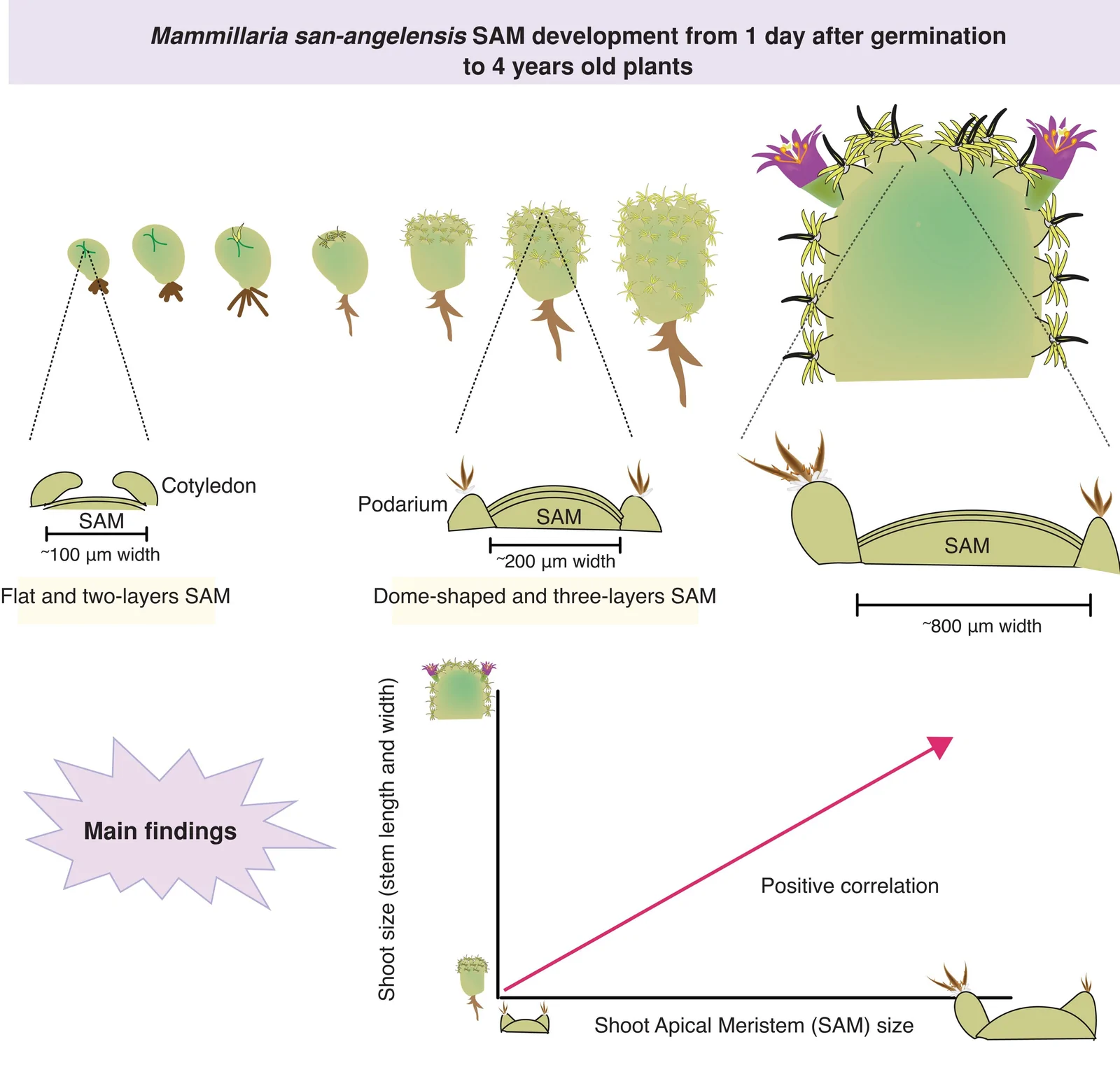

## INTRODUCTION

The use of *Arabidopsis thaliana* as a model organism dates back to 1907, when Friedrich Laibach first documented it while searching for a plant with a small number of chromosomes (Meyerowitz, 2001). This marked the beginning of the development of seed stocks, which facilitated extensive research in plant biology (Koornneef and Meinke, 2010). Today, *A. thaliana* is the most studied plant, and much of our understanding of plant biology is attributable to research conducted on it, including the study of the shoot apical meristem (SAM).

The *A. thaliana* SAM has a dome-shaped tip at the stem and generates all aerial organs (stems, leaves and flowers) throughout the lifespan of the plant (Kuhlemeier, 2007). The SAM is formed and organized during embryogenesis, and post-embryonic development occurs thanks to axillary meristems (AMs) in the leaf axil, allowing for branching (Shi and Vernoux, 2019). AMs are structurally and functionally similar to embryonic SAMs. During reproductive transition, the SAM is transformed into the inflorescence meristem (IM), which generates floral meristems (FMs) that produce floral organs, including sepals, petals, stamens and carpels (Shi and Vernoux, 2019). The relationship between the SAM and organ traits has been poorly described. However, there are few studies that describe the allometry between SAM parameters, such as cell size, number of cells, SAM width and thickness and the organ traits it generates, such as flowering time, number of leaf nodes, leaf area, stem diameter, height and seed mass (Leiboff *et al.*, 2015; Schnablová *et al.*, 2017). Besides, these correlations show a functional link influenced by phylogenetic history and parallel evolution (Schnablová *et al.*, 2017). Likewise, some architectural traits of plants, such as height and width, are influenced by proliferation and differentiation in the SAM and are correlated with various quantitative measurements of SAM morphometric parameters (Narnoliya *et al.*, 2019).

The *A. thaliana* SAM is organized into distinct zones and domains. The central zone (CZ), located at the centre of the SAM, is where slow division occurs and contains stem cells. Within the CZ is placed the organizing centre (OC), which is a niche that sustains stem cells. The peripheral zone (PZ) flanks both sides of the CZ, where cells divide rapidly and will originate leaf primordia. Finally, the rib zone (RZ) is positioned below the CZ and is responsible for developing the pith and vascular structures of the stem (Dodsworth, 2009; Galli and Gallavotti, 2016; Shi and Vernoux, 2019). Besides, the SAM consists of three cell layers: L1, the epidermal layer; L2, the sub-epidermal layer; and L3, the corpus. Layers L1 and L2 form the tunica and divide anticlinally, whereas L3 is a multilayer group of cells located below the tunica and can divide into all planes (Traas and Vernoux, 2002).

Under a microscope, the SAM seems simple, but a complex genetic network produces different cell identities. The first genes identified to be involved in SAM function were *AGAMOUS* (*AG*) and *KNOTTED* (*KNT*) in maize (Hake *et al.*, 1989; Yanofsky *et al.*, 1990). Other important genes, such as *WUSCHEL* (*WUS*), *CLAVATA3* (*CLV3*), *SHOOT MERISTEMLESS* (*STM*) and members of the CLASS III HOMEODOMAIN LEUCINE ZIPPER (HD-ZIPIII) transcription factors, participate in meristem size; the loss of function of these genes causes defects in SAM development (Mandel *et al.*, 2016; Shimotohno, 2022). Progress on study of the SAM in *A. thaliana* has been particularly rapid, mainly owing to the large number of available mutants, new mapping strategies, transformation protocols, reporter lines, the whole-genome sequence and affordable sequencing technologies.

Attributes of the SAM include its participation in phyllotaxy, leaf types and stem size (Barton, 2010). The SAM size measured by height by width is approximately 40 μm × 60 μm in *A. thaliana* and *Antirrhinum majus*, and the biggest one in another model plant is *Zea mays*, at 200 μm × 200 μm (Barton, 2010). In most seed plants, the SAMs have a constant size ranging from 100 to 300 μm (Mauseth, 1988). However, limiting studies to a few plant species reduces our understanding of plant diversity and its adaptations.

In recent years, new model systems have emerged for various plant groups, particularly among eudicot angiosperms, which now include representatives from ten families (Chang *et al.*, 2016). However, no established plant model for the Cactaceae family has been used to study SAM developmental biology. Only a few significant studies have examined determinate primary root growth in various cactus species, including *Pachycereus pringlei* (Shishkova *et al.*, 2013; Rodriguez-Alonso *et al.*, 2018). However, most research on cacti has focused primarily on phylogeny, phylogeography and population genetics (Franco *et al.*, 2022). Over the decades, essential descriptions of SAM anatomy across various cactus species have helped to elucidate how SAM gigantism occurs and how the SAM gives rise to aerial organs (Boke, 1944, 1952, 1953, 1955, 1958; Mauseth, 1978, 2004; Miravel-Gabriel *et al.*, 2024). Nevertheless, the genetic basis of SAM development in the family Cactaceae has been largely overlooked. Mauseth (2004) was the first to propose that genes controlling giant SAMs in cacti might be similar to those in *A. thaliana*, such as *WUS*, *CLV3* and *STM*. We recently proposed how the genetic mechanisms known in model plants might be responsible for the diversity of growth patterns, phyllotaxy and gigantism in cacti (López-Ruiz *et al.*, 2025). Members of the Cactaceae family, such as *Mammillaria*, possess specialized structures known as areoles and podaria. These structures are considered axillary meristems and leaf bases, which can give rise to spines, trichomes, flowers and even roots. Both areoles and podaria originate from the SAM (Boke, 1944, 1952, 1953, 1955, 1958; Mauseth, 2017). Although numerous studies have examined their anatomy, the genetic basis of their development remains unclear.

The Cactaceae are a large family of plants native to the Americas, consisting of ∼150 genera and 1851 species belonging to the order Caryophyllales (Korotkova *et al.*, 2021). Mexico has the most significant number of described Cactaceae species in the world. Studying these plants is vital owing to their adaptations to arid and semi-arid environments. Cacti exhibit essential characteristics such as succulence and Crassulacean acid metabolism (CAM) to cope with low water availability (Mauseth, 2018; Flores *et al.*, 2022). Furthermore, the presence of areoles is a unique structure found only in the Cactaceae family, which enables these plants to produce spines, trichomes, branches and flowers (Boke, 1958, 1980; Mauseth, 2017*a*, *b*).

We are currently developing the Mexican cactus *Mammillaria san-angelensis* as a model system to study the evolutionary novelties found in members of the Cactaceae family adapted to arid and semi-arid environments. Such evolutionary novelties include succulence, podarium and areole development, CAM photosynthesis and SAM gigantism.

*Mammillaria san-angelensi*s is a globose cactus that reaches 6–12 cm in height and 4–7 cm in width, maturing in ∼4 years (Sánchez-Mejorada, 1981). Its hermaphroditic flowers are arranged in a crown-like pattern and, like other *Mammillaria* species, it exhibits dimorphic areoles, with spines and trichomes on the upper side and flowers at the base. *Mammillaria san-angelensis* was endemic to the Pedregal de San Ángel area in Mexico City, now extinct, but conservation efforts have allowed for its propagation for research and morphological studies (Castillo-Argüero *et al.*, 2004). This cactus has several advantages, including a shorter time to reach sexual maturity than other cacti, a smaller size than other members of the Cactaceae family, ease of propagation and the fact that we are currently assembling its genome. Several developmental studies have focused on root architecture in *M. san-angelensis*, including identifying *PLETHORA* (*PLT*) genes (Parada-Ponce, 2004; González-García *et al.*, 2011; Cisnero-Lemus, 2024; Torres-Flores, 2025) and flower allometry (Rosas *et al.*, 2022). We are now interested in investigating the morphogenesis of the SAM in Cactaceae using *M. san-angelensis* as a model system.

Research has indicated a positive correlation between SAM size and stem diameter in various cacti species at the adult stage (Mauseth, 2004). We are now investigating whether this correlation also exists during the early stages of plant development by incorporating new measurements of both the SAM and the overall size of the plant. At the same time, we aimed to describe the morphoanatomy of three meristem types: the vegetative SAM, the floral meristem and the meristem of the areole of eight developmental stages of *M. san-angelensis*, ranging from 1 day post-germination to 4 years of growth. We analysed the anatomy of the cactus SAM and its relationship to plant morphology, specifically stem length and width, to test whether changes in SAM size and shape can explain the increase in stem size and allometry. This comparison seeks to generate hypotheses regarding how the Cactaceae family can develop diverse shapes and adapt to extreme environments.

## MATERIALS AND METHODS

### Seed obtention and growth conditions

*Mammillaria san-angelensis* was an endemic plant found in a natural area of Mexico City known as Pedregal de San Angel. However, it is now considered extinct in its natural habitat since 1985 (Martínez-Vázquez and Rubluo, 1989). The Botanical Garden at Universidad Nacional Autónoma de México (UNAM) has made significant efforts to propagate this species using mother plants sourced from its original habitat. Jeronimo Reyes, the curator of the Crassulaceae collection at the Botanical Garden, provided the seeds used for propagation. *Mammillaria san-angelensis* has been propagated under a Organization for the Conservation of Wildlife (Unidad de Conservación para la Vida Silvestre, UMA).

Seeds of *M. san-angelensis* were soaked in water for 48 h. After soaking, they were placed in a sealed translucent plastic container to maintain humidity. The seeds were covered with a mixture consisting of 40 % black soil, 40 % tepojal (volcanic pumice) and 20 % tezontle (volcanic rock). This is a commercial substrate typically used for cacti. The container was then placed in a growth chamber set at 28 °C, with a photoperiod of 12 h of light (112 µmol m^−2^ s^−1^) and 12 h of darkness (Percival Scientific, Iowa, USA). The visibility of the radicle indicated seed germination. Plants that were 4 years old were grown in a greenhouse. The change from a growth chamber to greenhouse conditions is required because plants need warmer, lighter conditions to flower.

### Morphological characterization

Eight developmental stages of growth were determined by the morphological traits of the plantlets and the number of days after germination (dag). Stage 1 was defined as 1 dag, characterized by closed cotyledons and no evidence of areoles. Stage 2, at 3 dag, is marked by a slight opening of the cotyledons, with no signs of areole formation. In stage 3, at 7 dag, the spines start to be evident. Stage 4, at 15 dag, shows at least two visible areoles. At stage 5, 30 dag, the stem is noticeable, with the hypocotyl being about one-third the height of the plantlet. At 90 dag, stage 6 is characterized by the areoles covering half of the plant body. By 1 year of age, the plants exhibit areoles with more rigid and longer spines, and the typical pattern of spines for the species is clear. At 4 years old, plants reach maturity and are capable of flowering. The observations were made using a digital Monozoom microscope, and the images were edited with Photopea (v.5.4) and Adobe Illustrator (v.29.8.5).

The measurements were made with ImageJ (v.1.54g), using the scale of each image as reference. For plantlets aged 1–15 dag (stages 1–4), the measurements recorded were the length and width of the plantlet. In these stages, the length was measured from the hypocotyl to the base of the cotyledons, and the width was measured at the widest part of the plantlet. For stages 5–7 (30 dag, 90 dag and 1 year), the length was divided into two parts: the hypocotyl, which is identified as being free of spines; and the stem, which features areoles. For stage 8 (4 years old), the length measurement included only the stem, which is characterized by being completely covered with areoles. The length was measured from the beginning of the root to the apex. In all cases, the width was measured at the widest part of the plant.

### Histological characterization

For the anatomical analysis, two protocols were applied to eight developmental stages. Stages 1, 2, 3, 4, 5 and 6 correspond to 1, 3, 7, 15, 30 and 90 dag, respectively. Stages 7 and 8 correspond to 1 and 4 years after germination (yag). Only the apical part of the stem was collected for this study. The first protocol used Paraplast (Leica, Germany) for embedding, whereas the second protocol used HistoResin (Leica, Germany).

For the Paraplast method, samples were fixed in formaldehyde–acetic acid–alcohol and dehydrated for >24 h using a progressive series of *tert*-butyl alcohol (30, 50, 70, 80, 95 and 100 %) (Sandoval-Zapotitla *et al*., 2005). A vacuum pump was used for 15 min following each change in concentration. After dehydration, the samples were embedded in Paraplast blocks using a Paraplast infiltrator (Leica, HistoCore Arcadia H-C) set at 60 °C. The samples were sectioned longitudinally to a thickness of 7 μm using a LEICA RM2125 rotary microtome. After sectioning, the slides were stained with alcoholic Safranin and Fast Green, then mounted with two to four drops of resin (Sandoval-Zapotitla *et al*., 2005).

For the protocol using Historesin, all samples were fixed for 24 h in formaldehyde–acetic acid–alcohol. After fixation, the samples were dehydrated in a series of ethanol concentrations: 30, 50, 70, 96 and 100 %. A vacuum pump was used for 15 min following each change in concentration. The samples were then infiltrated with resin at various concentrations (30, 50, 70, 90 and 100 %), with a vacuum applied for 15 min after each change. Finally, the samples were embedded in resin using silicone moulds and an ultraviolet lamp until the resin hardened. The samples were then cut longitudinally on a rotating microtome to a thickness of 7 μm. The sections were stained with Toluidine Blue and mounted with synthetic resin for observation.

For both procedures, photomicrographs were captured under a bright-field microscope (Zeiss Axioscope) equipped with a digital camera (PANASONIC imxCMOS), using the Rising View of AmScope software. The photographs were then edited with Photopea (v.5.4) to eliminate the background noise.

The SAM measurements included five traits: dome width, which is the length from cotyledon to cotyledon (stage 1 of development), and in later stages, the length from podarium to podarium; the height of the dome; the perimeter of the dome; and the area and perimeter of the SAM. The area and perimeter of the SAM include not only the dome but also the meristematic zone, which is characterized by the presence of small, highly nucleated cells.

### Scanning electron microscopy

For scanning electron microscopy, samples from plants at 1, 3, 7 and 15 dag and the spines of adult plants were fixed using formaldehyde–acetic acid–alcohol for 24 h at room temperature. The tissue samples were then washed three times with distilled water and dehydrated through a graded series of ethanol concentrations (10, 20, 30, 40, 50, 60, 70, 90 and 100 %) over the course of 24 h. Subsequently, the samples were dried by critical-point drying with liquid carbon dioxide. Finally, the tissues were coated with gold for 2 min on a sputter coater (model Q150R, Quorum, Laughton, UK). The prepared samples were examined and photographed using a Hitachi SUI510 scanning electron microscope (Tokyo, Japan) operating at an accelerating voltage of 10–15 kV.

### Data visualization and statistical analysis to explore the correlation between SAM and plant traits

All analyses were performed in Python 3.10.16 using NumPy 2.2.2, pandas 2.2.3, SciPy 1.15.1, scikit-posthocs 0.11.4, seaborn 0.13.2 and matplotlib 3.10.0. Across stages 1–8, the number of biological replicates ranged from 8 to 20 for plantlet measurements and from 3 to 7 for SAM measurements. The measurements for plant traits are displayed in Supplementary Data Table S1. Floral meristem measurements (stage 8 only) were obtained from three biological replicates. Given that histological slides showed varying measurements depending on the SAM dome sections, only the highest value was used in the statistical analysis (Supplementary Data Table S2).

For trait visualization of plantlet and SAM measurements, we created box plots showing the distribution of each trait. To test for overall differences among stages 1–7, we applied the Kruskal–Wallis test. The adult stage (stage 8) was excluded from this analysis because its values were higher (≥10 times) compared with the juvenile stages (1–7). When the Kruskal–Wallis test was significant (α = 0.05), pairwise *post hoc* comparisons were performed using Dunn’s test with the Holm adjustment.

Because the allometry between SAM and organ traits has been studied previously, we evaluated the relationships between plantlet and SAM variables. For that, we computed the mean value per stage for each variable. Because plantlet and SAM measurements were obtained from different individuals and could not be paired one-to-one, correlations were calculated based on stage means. Spearman correlation coefficients were estimated using a bootstrap approach (1000 iterations). We report the bootstrap mean ± s.d. in a heatmap, marking correlations whose 95 % bootstrap confidence interval did not cross zero. We also calculated, for each variable pair, the percentage of valid bootstrap iterations yielding Spearman correlation coefficients of >0.80 and present these frequencies as a heatmap. Additionally, the plantlet and SAM variables were log_1_  _+_  *_x_*-transformed, and their mean per stage were used to generate scatterplots for every unique variable pair. Each scatterplot was coloured by developmental stage and included a fitted linear regression trend line. The coefficient of determination (*R*^2^) was annotated in the top corner of each plot (Supplementary Data Fig. S1).

For adult floral meristem variables (stage 8), the mean value of each trait was calculated, and bar plots of these means were generated. Individual sample values (*n* = 3) were overlaid as coloured points.

## RESULTS

### General description of the model system

**Fig. 1.**
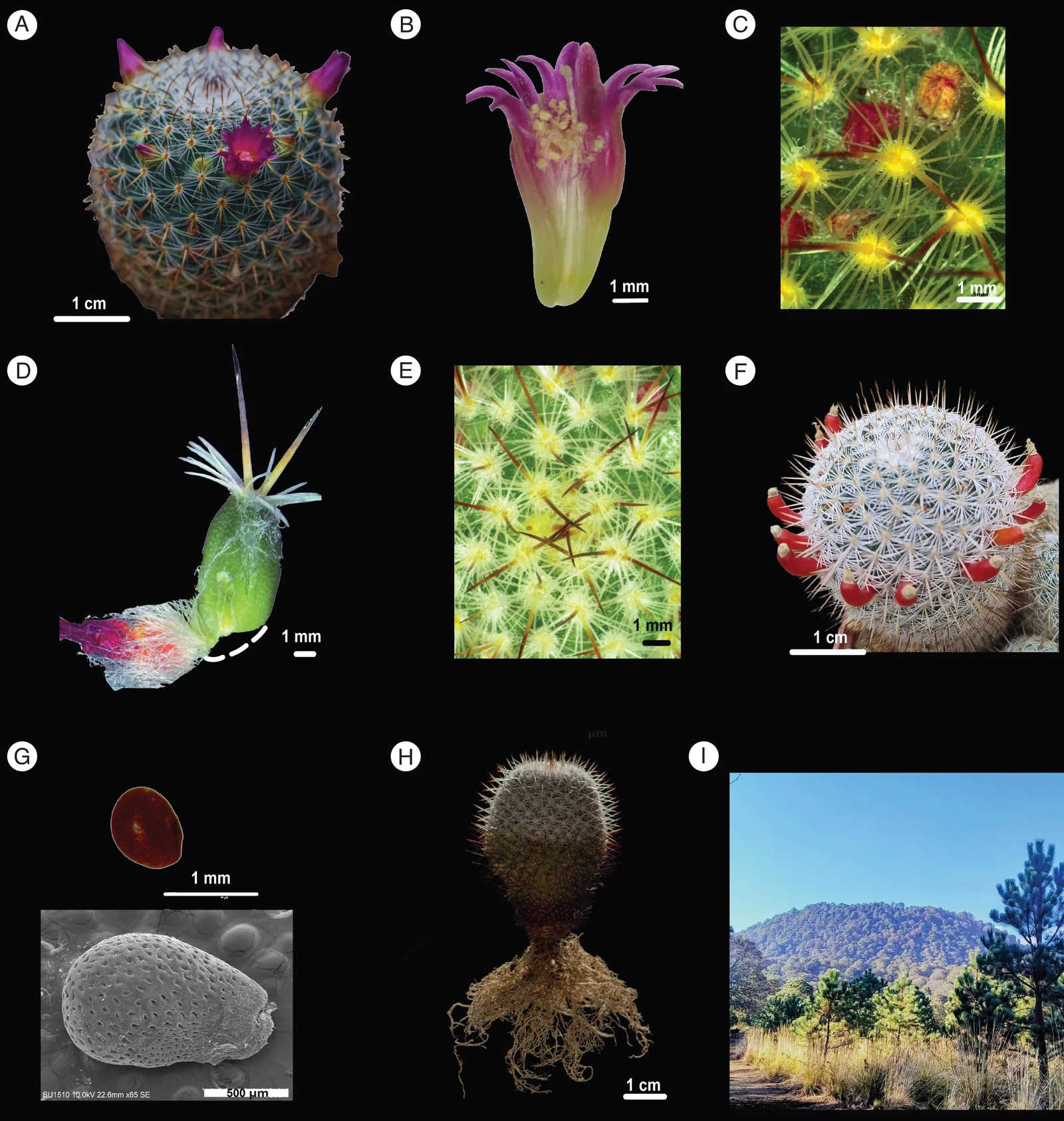
Main features of *Mammillaria san-angelensis*, which was used as a model system to study the development of the shoot apical meristem (SAM). (A) An adult individual, 4 years old, in flower. (B) A close-up of a flower displaying its radial symmetry. (C) A zoomed-in view of the stem, highlighting the spines and flowers. (D) A dimorphic areole, characteristic of producing flowers at the base and spines at the upper part of the podarium. The dashed line indicates the surrounding tissue that is naturally present in the stem, connecting the podarium with the flower. (E) An aerial view of the shoot. (F) The fruits of *M. san-angelensis*. (G) Seeds. (H) The root system. (I) *Mammillaria san-angelensis* is native to the San Angel natural reserve (REPSA), which was shaped by the eruption of the Xitle volcano.

Our model system is *M. san-angelensis*, a globose cactus that typically grows to a height of ∼6–12 cm and a width of 4–7 cm, reaching maturity in ∼4 years (Fig. 1A). The flowers of this cactus are hermaphroditic (Fig. 1B) and are arranged around the plant like a crown (Fig. 1A). *Mammillaria san-angelensis*, like other members of the *Mammillaria* genus, displays dimorphic areoles, that is, the upper side of the tubercle or podarium produces spines and trichomes, while at the base of the podarium, flowers grow (Fig. 1C, D). The apex is sunken and covered with spines and trichomes that protect the SAM (Fig. 1E). Each fruit can produce ≤20 seeds, each measuring ∼1 mm long (Fig. 1F, G). The seeds are oval in shape, with a concave surface and isodiametric cells (Parada-Ponce, 2004; Fig. 1G). The roots of *M. san-angelensis* are highly branched (Fig. 1H) and shallow.

*Mammillaria san-angelensis* was an endemic species found in the Pedregal de San Ángel (Reserva del Pedregal de San Angel, REPSA) area of Mexico City (Fig. 1I). It grew naturally on the volcanic rock produced by the eruption of the Xitle volcano, which occurred ∼1670 years ago (Siebe, 2009). This species is now considered extinct in its natural habitat owing to habitat loss and extraction activities. However, significant conservation efforts by the Botanical Garden of the Institute of Biology at UNAM have enabled the propagation of this cactus, leading to the availability of seeds and plants for conservation, propagation, research and, particularly in this work, for morphological studies.

### Establishment of the developmental stages

**Fig. 2.**
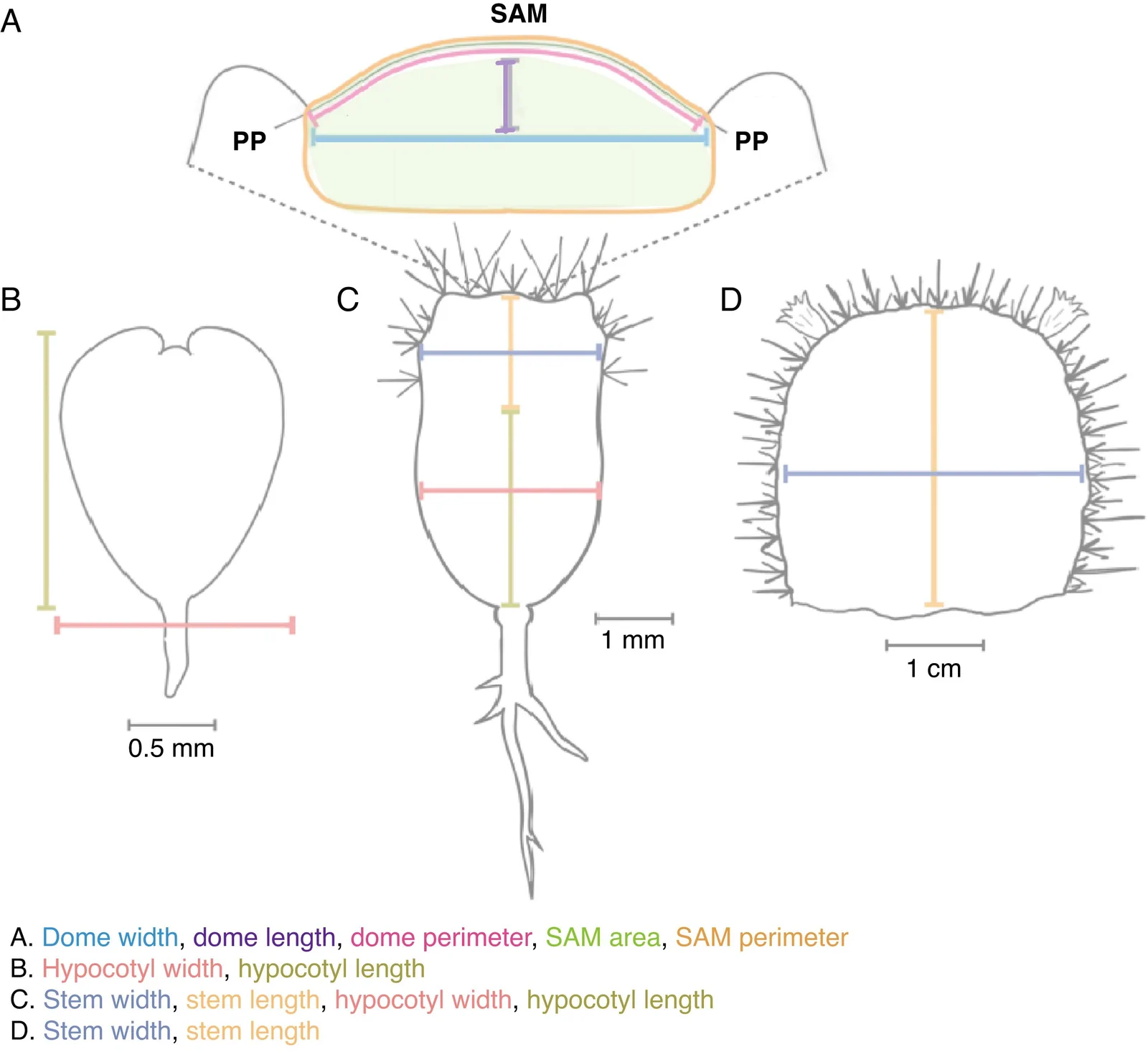
Schematic representation of the shoot apical meristem (SAM) and the whole plant in different stages. Measurements of *Mammillaria san-angelensis* were taken from seedlings to adult individuals. (A) The SAM was evaluated based on its width, length, perimeter, and area of the SAM dome as indicated in the image using different colours. (B) For plantlets [1–15 days post-germination (dag), stages 1–4], only the width and length of the hypocotyl were measured. (C) In plantlets in stages 5–7, measurements included stem width and length, in addition to hypocotyl length and width. (D) For stage 8, the measurements include only stem width and length.

Seeds of *M. san-angelensis* were soaked and germinated in soil, with germination defined by the emergence of the radicle. The developmental stages were distinguished by measuring four aerial traits, including hypocotyl, stem length and width, and five SAM measurements (Fig. 2). The first four stages (1–15 dag) show only the presence of the hypocotyl without any stem (Fig. 2B). In stages 5–7 (from 30 dag to 1 yag), stems are now visible, emerging at stage 5, characterized by the growth of areoles that cover the stem (Fig. 2C). By the final stage, 4 yag, the stem is completely covered with areoles, and the hypocotyl disappears (Fig. 2D). The SAM was measured and divided into two main parts: the dome and the whole meristematic zone, which includes the proliferative cells located immediately beneath the dome (Fig. 2). The measurements of the SAM dome include width, length and perimeter, and the meristematic zone was assessed based on its perimeter and area, as shown in Fig. 2A.

### Morphoanatomical features of each developmental stage

**Fig. 3.**
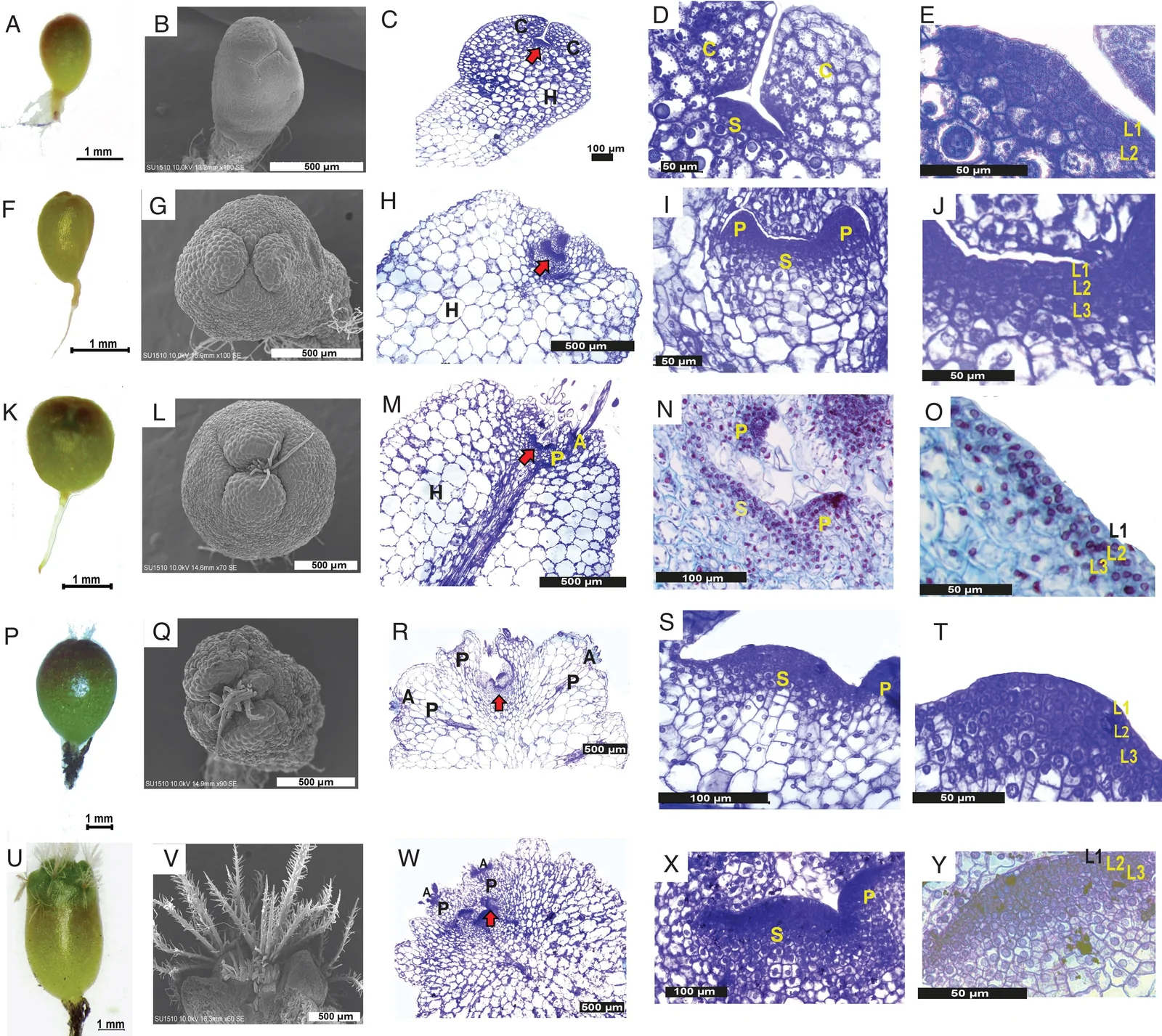
Developmental stages of *Mammillaria san-angelensis* plantlets. (A–E) Stage 1, 1 dag. (F–J) Stage 2, 3 dag. (K–O) Stage 3, 7 dag. (P–T) Stage 4, 15 dag. (U–Y) Stage 5, 30 dag. (A, F, K, P and U) Plantlets observed under stereoscopic microscopy. Scale bars: 1 mm. (B, G, L, Q and V) Scanning electron microscope images. (C, H, M, R and W) panoramic histological images at ×4 magnification. Scale bar: 100 or 500 μm. The red arrow indicates the shoot apical meristem (SAM). (D, I, N, S and X) Close-ups showing the SAM at each stage (×40). Scale bars: 50 or 100 μm. (E, J, O, T and Y) Close-ups displaying the SAM layers labelled as L1, L2 or L3. Abbreviations: A, areole; C, cotyledon; H, hypocotyl; L, layer; P, podarium; S, shoot apical meristem. Stage 1 (1 dag; A–E) is characterized by closed cotyledons with no areoles. At this stage, the SAM is located between the cotyledons and appears slightly flattened, consisting of only two cellular layers. In stage 2 (3 dag; F–J), the podaria are evident at both sides of the SAM, and three SAM layers are formed. In stage 3 (7 dag; K–O), the spines start to emerge, and the SAM is ∼100 μm. In stages 4 and 5 (15 and 30 dag; P–T and U–Y), the spines cover the apex of the plantlet, and the SAMs acquire a dome-shaped form.

The first stage was established 1 dag (Fig. 3A, B), during which the plantlet exhibits a hypocotyl measuring 1.464 (s.d. ±0.325) mm in length and 0.979 (s.d. ±0.142) mm in width (Table 1). This stage is characterized by closed cotyledons, with no evidence of areoles. The SAM has a width between the cotyledons of 102.007 (s.d. ±30.178) µm; the dome length is barely perceptible (Table 2) and, in some cases, it is flat or concave (Fig. 3C, D). At this stage, only two layers in the SAM are distinguished (Fig. 3E).

**Table 1.** Allometry of plantlets and plants of *Mammillaria san-angelensis*.

Developmental stage	Hypocotyl width (mm)	Hypocotyl length (mm)	Stem width (mm)	Stem length (mm)	Total length (mm)
1 (1 dag)	0.979 ± 0.142	1.464 ± 0.325	NA	NA	1.464 ± 0.325
2 (3 dag)	1.417 ± 0.101	1.687 ± 0.193	NA	NA	1.687 ± 0.193
3 (7 dag)	1.708 ± 0.246	2.018 ± 0.290	NA	NA	2.018 ± 0.290
4 (15 dag)	1.885 ± 0.406	2.365 ± 0.475	NA	NA	2.365 ± 0.475
5 (30 dag)	2.413 ± 0.169	2.235 ± 0.522	2.397 ± 0.235	1.793 ± 0.356	4.028 ± 0.625
6 (90 dag)	1.401 ± 0.349	1.347 ± 0.449	2.044 ± 0.248	2.994 ± 0.322	4.319 ± 0.457
7 (1 yag)	1.64 ± 0.359	0.837 ± 0.246	3.33 ± 0.732	4.452 ± 1.125	5.289 ± 1.065
8 (4 yag)	NA	NA	28.625 ± 2.446	28 ± 2.777	28 ± 2.777

Plant measurements (mean and s.d.) were taken in the eight different developmental stages. Abbreviations: dag, days after germination; NA, not applicable; yag, years after germination.

**Table 2.** Allometry of the shoot apical meristem of *Mammillaria san-angelensis*.

Developmental stage	Dome width (µm)	Dome length (µm)	Dome perimeter (µm)	SAM area (µm^2^)	SAM perimeter (µm)
1 (1 dag)	102.007 ± 30.178	10.787 ± 10.337	85.192 ± 67.916	3021.12 ± 681.322	257.455 ± 41.26
2° (3 dag)	94.156 ± 23.718	22.1 ± 3.225	113.58 ± 27.332	2972.713 ± 792.843	237.847 ± 57.339
3 (7 dag)	140.232 ± 17.0308	21.68 ± 0.487	155.412 ± 15.506	4385.775 ± 831.505	314.56 ± 36.408
4 (15 dag)	128.095 ± 23.935	28.525 ± 7.4254	144.68 ± 24.965	3689.74 ± 894.957	291.202 ± 50.042
5 (30 dag)	136.964 ± 24.853	38.954 ± 3.354	162.304 ± 19.849	5361.455 ± 1185.704	324.0345 ± 61.563
6 (90 dag)	212.077 ± 34.022	32.253 ± 4.0311	226.137 ± 43.247	7970.7 ± 2576.882	470.363 ± 55.981
7 (1 yag)	224.657 ± 22.407	38.447 ± 5.078	241.853 ± 22.231	7739.253 ± 498.539	511.733 ± 42.303
8 (4 yag)	771.71 ± 64.533	95.81 ± 36.445	823.536 ± 75.362	58 391.777 ± 6139.436	1623.787 ± 104.753
Floral meristem	135.743 ± 3.069	30.537 ± 13.238	154.4 ± 15.321	4087.953 ± 1366.976	296.513 ± 18.013

Plant measurements (mean and s.d.) were taken in the eight different developmental stages. Abbreviations: dag, days after germination; yag, years after germination.

At the second stage, which occurs at 3 dag, the cotyledons show a slight opening, but there are no signs of areole formation (Fig. 3F, G). The hypocotyl begins to enlarge and thicken, measuring 1.687 (s.d. ±0.193) mm in length and 1.417 (s.d. ±0.101) mm in width (Table 2; Fig. 3F). Anatomical slides show that, adjacent to the SAM, small meristematic bumps, referred to as podaria, start to emerge (Fig. 3H, I). These bumps will eventually develop into the podarium, from which spines, trichomes and incipient leaves will form. Although the SAM has not increased in size, its dome length is now more prominent, measuring 22.1 (s.d. ±3.225) mm in length (Table 2). At this stage, three SAM layers are visible (Fig. 3J).

On 7 dag, the third stage was established (Fig. 3K–O). In this stage, the most prominent feature is the development of the areole, the distinctive trait of the Cactaceae family. The scanning electron microscopy and histological analysis show the presence of spines for the first time. Besides, all the SAM traits evaluated (width, length, area and perimeter) are bigger compared with the previous two stages (Table 2; Fig. 3K).

In the fourth stage, which was recognized at 15 dag, at least two enlarged podaria with areoles displaying spines are observed (Fig. 3P–R). The hypocotyl measures 2.365 (s.d. ±0.475) mm in length, and at this stage, it will be the last to show only the hypocotyl, because the stem starts to grow (Table 1). Additionally, the SAM does not show an increase in width compared with the previous stage, but it does show growth in length (Table 2).

In the fifth stage, at 30 dag, the stem becomes noticeable for the first time (Fig. 3U**)**, with the hypocotyl measuring more than half the total height of the plantlet (Table 1). The presence of areoles indicates the distinction between the stem and the hypocotyl. The total length, including the hypocotyl and stem, is ∼4.028 (s.d. ±0.625) mm. At this stage, the SAM shows evident changes in both dome length and area (Table 2).

**Fig. 4.**
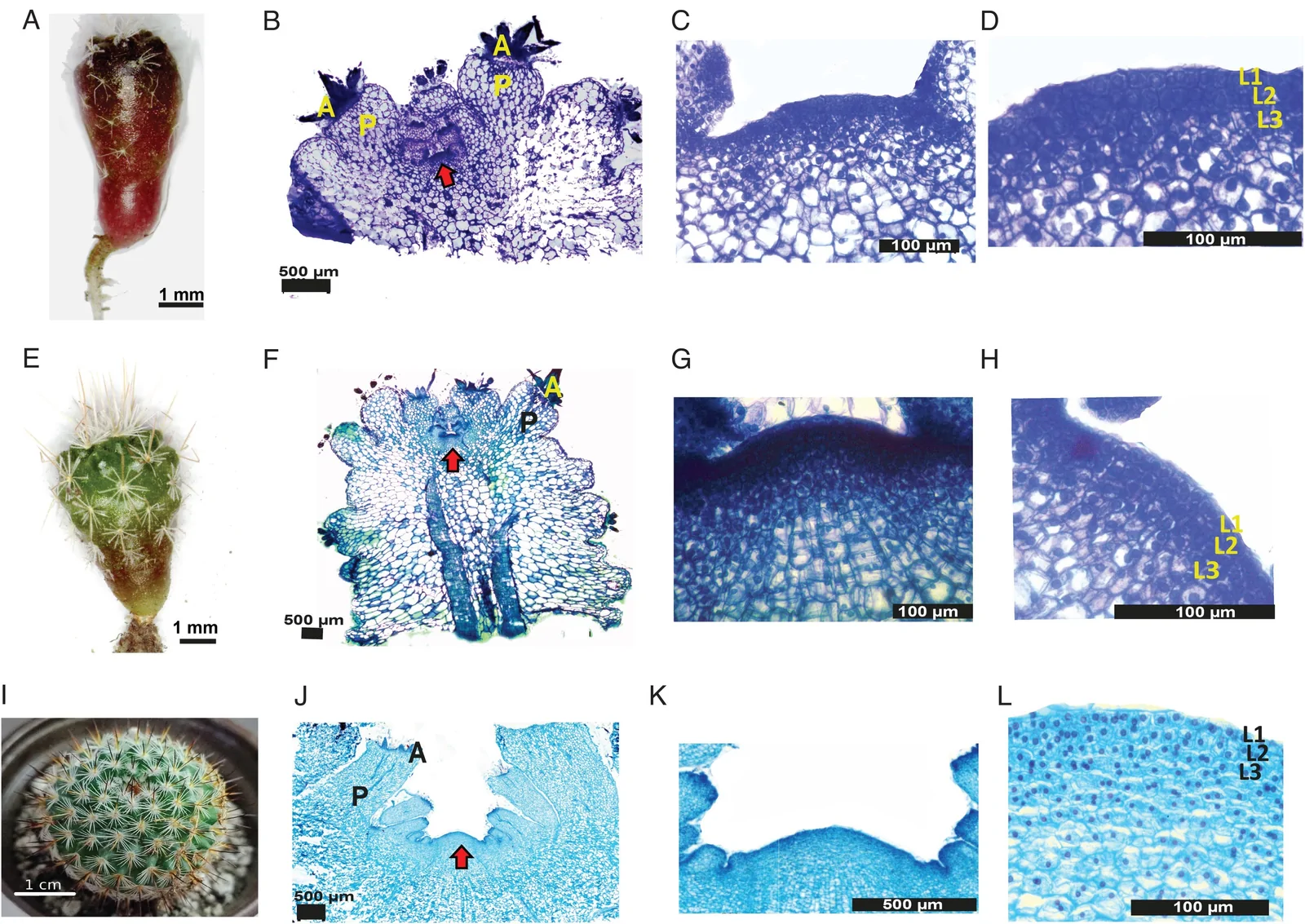
Developmental stages of *Mammillaria san-angelensis* plantlets. (A–D) Stage 6, 3 months after germination. (E–H) Stage 7, 1 year after germination (yag). (I–L) Stage 8, 4 yag. (B, F and J) Panoramic histological images at ×4 magnification. Scale bars: 500 μm. The red arrow indicates the shoot apical meristem (SAM). (C, G and K) Close-ups show the SAM at each stage (×40). Scale bars: 100 μm in C and G and 500 μm in K. (D, H and L) Close-ups displaying the SAM layers labelled as L1, L2 or L3. Scale bars: 100 μm. Abbreviations: A, areole; P, podarium.

At 90 dag, the sixth stage is characterized by areoles covering two-thirds of the plant body (Fig. 4A). The hypocotyl length begins to decrease, while the stem length increases. The podarium covers the vegetative apex and exhibits sclerified spines (Fig. 4B). The SAM shows a significant increase in all measurements taken, except for dome length (Fig. 4C; Table 2). The dome has a width of 212.077 (s.d. ±34.022) µm, with a length of 32.253 (s.d. ±14.031) µm. Additionally, the meristematic area also demonstrates an increase, measuring 7970.7 (s.d. ±2576.882) µm^2^.

By 1 year of age, the seventh stage of *M. san*-*angelensis* development is established. At this stage, the plants exhibit areoles with longer and more rigid spines, and the characteristic spine pattern of the species is clearly visible (Fig. 4E). The hypocotyl is almost imperceptible, measuring only 0.837 (s.d. ±0.246) mm in length. The SAM has a width of 224.657 (s.d. ±22.407) µm and a length of 38.447 (s.d. ±5.078) µm (Fig. 4G, H).

At 4 years old, the plants were classified as the eighth stage, reaching maturity and flowering capability (Fig. 4I). The plantlets measure an average width of 28.625 (s.d. ±2.446) mm and a length of 28 (s.d. ±2.777) mm. The stem was fully covered with areoles, and the podaria reached their final length (Fig. 4I). At this stage, the SAM continued to grow, reaching an average width of 771.71 (s.d. ±64.533) µm and a length of 95.81 (s.d. ±36.445) µm (Fig. 4J, K). No more SAM layers are formed at this stage; only the established three layers are observed (Fig. 4L).

**Fig. 5.**
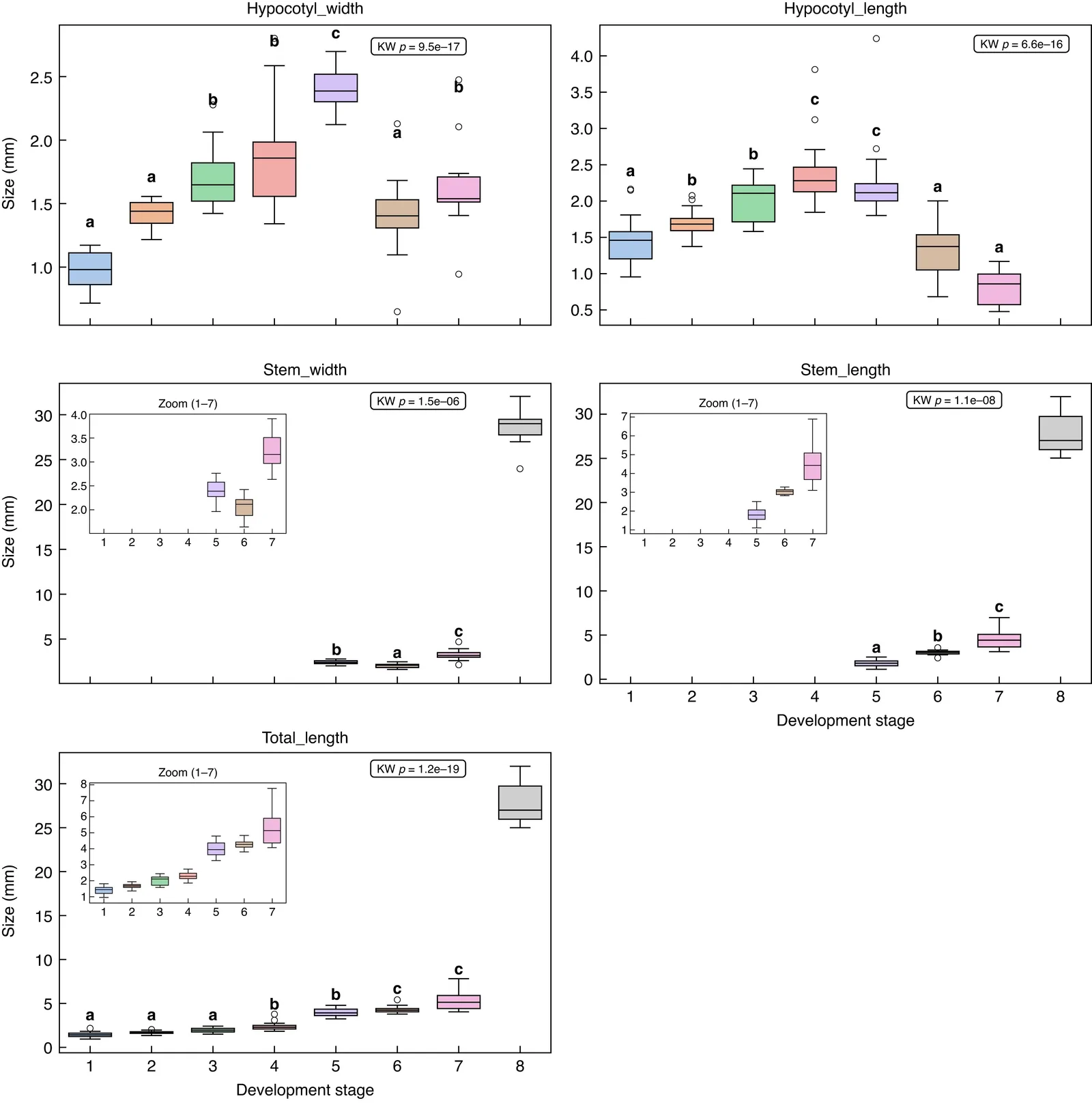
Quantitative development of *Mammillaria san-angelensis* plantlets and plants. Boxplot of the five plant measurements: hypocotyl width and length, stem width and length and total length (in millimetres) for the eight developmental stages of *M. san-angelensis*, as illustrated in Fig. 2. The developmental stages are defined as follows: stage 1, 1 dag; stage 2, 3 dag; stage 3, 7 dag; stage 4, 15 dag; stage 5, 30 dag; stage 6, 90 dag; stage 7, 1 yag; and stage 8, 4 yag. The inner rectangles zoom out the first seven stages for better comparability. Abbreviations: dag, days after germination; yag, years after germination.

**Fig. 6.**
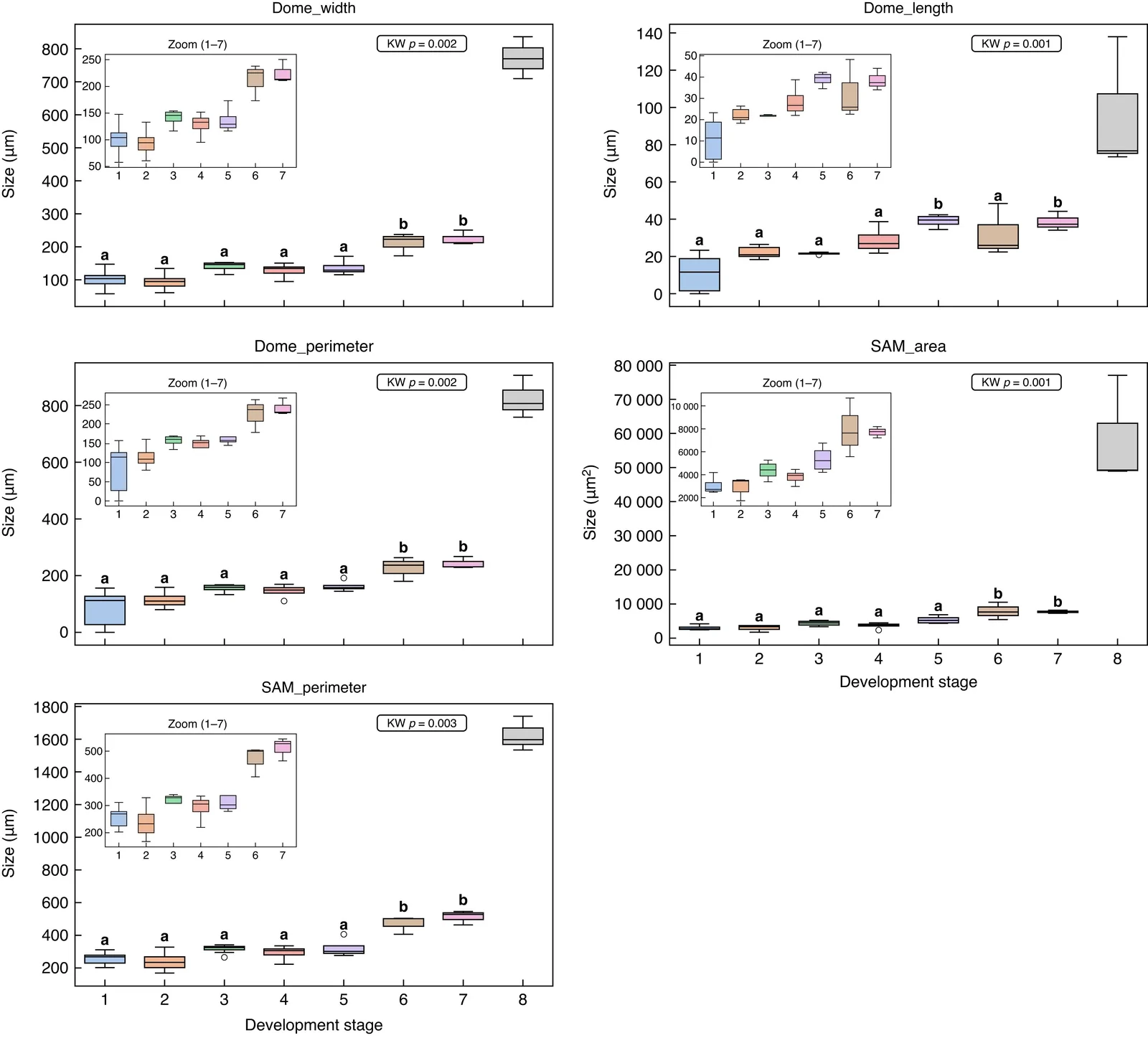
Quantitative shoot apical meristem (SAM) development of *Mammillaria san-angelensis*. Boxplot of the five SAM measurements: dome width and length, dome perimeter, SAM area and SAM perimeter (in micrometres) for the eight developmental stages of *M. san-angelensis*, as illustrated in Fig. 2. The developmental stages are defined as follows: stage 1, 1 dag; stage 2, 3 dag; stage 3, 7 dag; stage 4, 15 dag; stage 5, 30 dag; stage 6, 90 dag; stage 7, 1 yag; and stage 8, 4 yag. The inner rectangles zoom out the first seven stages for better comparability. Abbreviations: dag, days after germination; yag, years after germination.

When comparing all stages, it is evident that the hypocotyl length and width decrease over time until they eventually disappear (Fig. 5). In the first four stages, there are no data on the stem because it has not yet developed. The stem begins to grow as the podaria and their areoles form (Fig. 5). The SAM at stage 1 is either flat or minimally domed, as we observe in the dome length. The width of the SAM does not change significantly during the first two stages; the most notable changes occur at stage 6, reaching a maximum by stage 8. Additionally, the meristematic zone increases over time, as indicated by the area and perimeter of the SAM (Fig. 6).

### Comparison of the floral meristem and areole meristem in the dimorphic areole

**Fig. 7.**
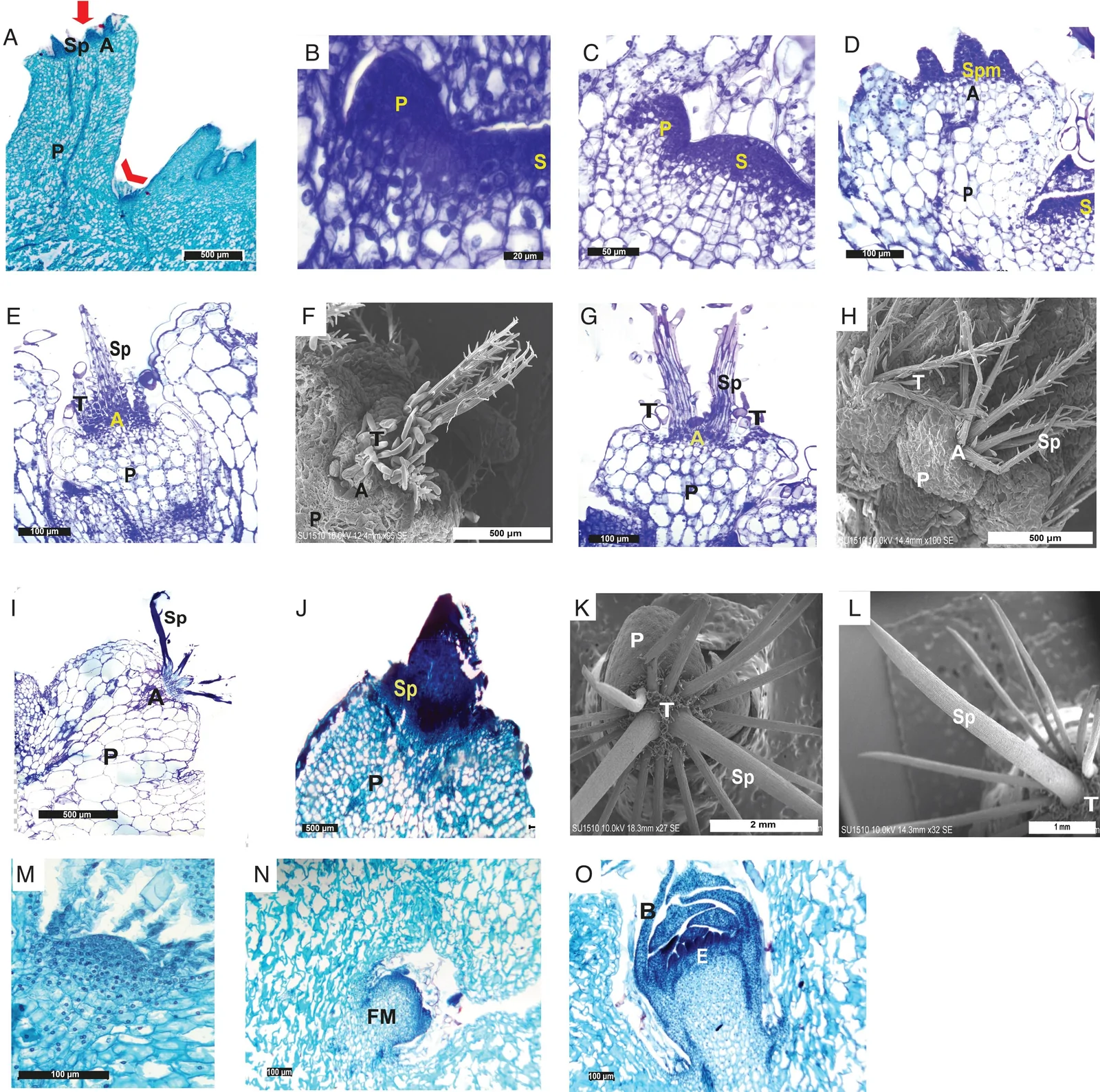
Development of spines and flowers in a dimorphic areole. (A) The aerial part of the podarium in an individual 4 years after germination (yag) displays a meristematic zone that produces spines and trichomes (red arrow). At the base of the podarium, a meristematic dome will give rise to a flower (arrowhead). (B–L) This series shows different stages of podarium formation leading to spine (Sp) development. (B and C) The presence of a meristematic bump that will produce the podarium (P). (D) The spiniferous meristem (Spm) and the aerial portion of the podarium are formed. (E and F) The spines and trichomes (T) are observed. (G and H) Spines are in an advanced developmental stage, showing ornamentations. (I) Spines are sclerified at 90 dag. (J) Areole of a 1-year-old individual showing sclerified spine. (K and L) Spines under scanning electron microscope at 4-year-old plants. (M–O) Initial stages of flower development. (M) Floral meristem (FM). (N) Elongation of the floral axis and lateral structures are covering the meristematic zone. (O) Formation of reproductive organs, such as the meristematic ring where stamen primordia (indicated in the figure by St) emerged, and tepals (Tp) are covering the floral meristem.

Members of the *Mammillaria* genus are characterized by having dimorphic areoles. This means that the summit of the podarium contains a meristematic zone that produces spines and trichomes, while at the base of the podarium a floral meristem develops (Fig. 7A).

The podarium is a structure with a debated origin (Ifrim and Feszt, 2022). It is typically considered a leaf base, but its exact origin has remained unclear. The podarium is formed by a meristematic bulge, flanking the SAM, that begins to elongate, resembling the leaf primordia observed in *A. thaliana* (Fig. 7B, C). The meristematic tissue of the developing podarium begins to be segmented, hence one portion is restricted to the apex, where the spines and trichomes will develop, and the other portion is located at the base, where the flowers will begin to develop, later generating a dimorphic areole (Fig. 7A). Once the podarium reaches ∼300 µm in length, the spiniferous meristem emerges (Fig. 7D). In Fig. 7E, the meristematic lumps that will originate spines are visible. The spiniferous primordia begin to elongate, and the spine ornamentations become apparent (Fig. 7F–H). Alongside the spines, trichomes can also be observed (Fig. 7E, F). Eventually, the spines undergo sclerification, losing their ornamentations as the plant reaches maturity (Fig. 7J–L).

**Fig. 8.**
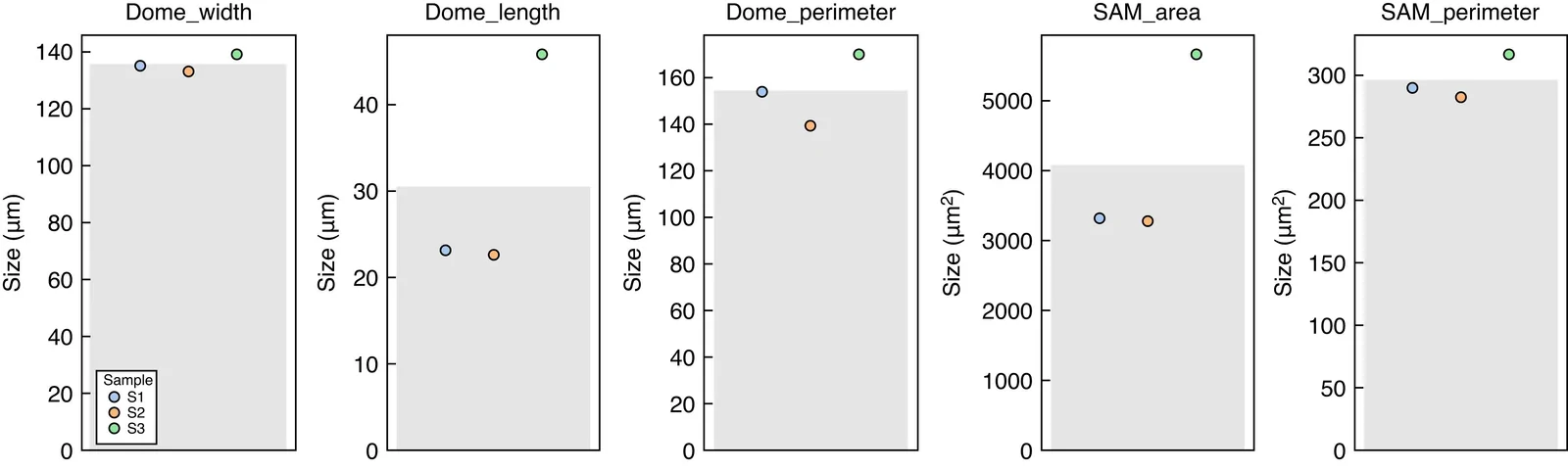
Five measurements were taken on the floral meristem from the 4-year-old adult plant apex. We used three biological samples, with each dot representing a measurement conducted on the floral meristem. The grey bar illustrates the mean value of the collected data. The *y*-axis represents the size (in micrometres) of the different measurements of the meristem flower.

The flower emerges from the base of the podarium as a meristematic bulge, which differs significantly in size from the vegetative meristem (Table 2; Fig. 8). The flower meristem is approximately six times shorter than the vegetative meristem, measuring 135.743 (s.d. ±3.069) µm in width and 30.537 (s.d. ±13.238) µm in length. It is positioned on the sides of the vegetative meristem (Fig. 4J). Although the complete developmental chronology of flower formation is not fully established, we show the initial stages of flower development (Fig. 7M–O), where the primordia of the stamens and bracts are observed.

### Multiple correlations between plant and SAM measurements

**Fig. 9.**
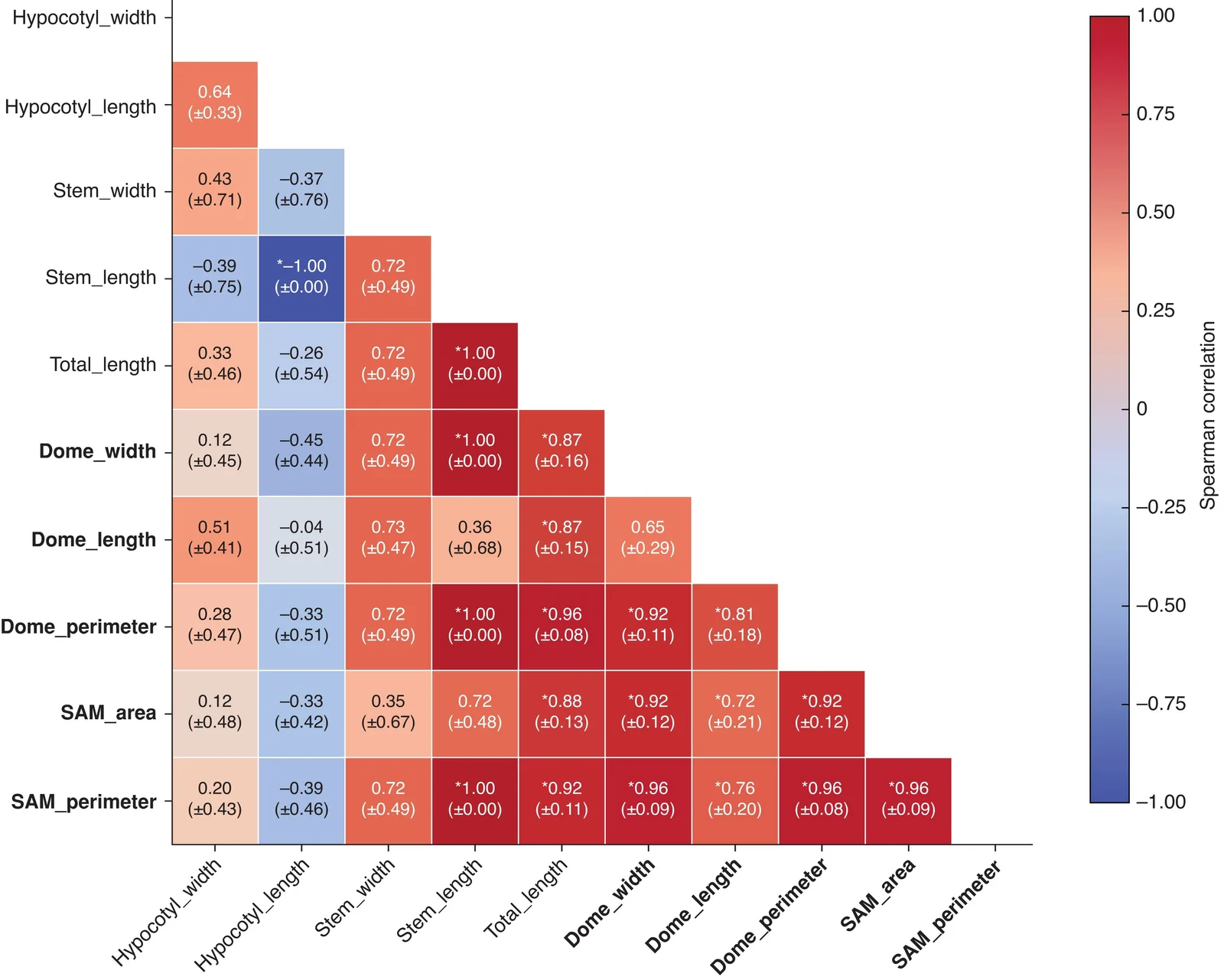
Allometric relationships between the shoot apical meristem (SAM) and the stem. There are multiple correlations among all the traits evaluated at the eight developmental stages of *Mammillaria san-angelensis*. Positive correlations are indicated by reddish colours, whereas negative correlations are shown by bluish colours. Asterisks indicate correlations whose 95 % bootstrap confidence interval did not include zero. Bold letters represent measurements taken in the SAM, and plain text denotes measurements for plant traits. The highest correlation was found between dome width of the SAM and stem length. Additionally, we recapitulate the previous findings of a positive correlation between SAM width and stem width.

Given that the allometry between the SAM and the aerial organs has been studied previously in cacti and other plants, we decided to perform multiple correlations between all the traits evaluated in *M. san-angelensis* (Fig. 9). The correlation between some traits is strong, as we can observe between the SAM traits such as dome width and length, perimeter and area and total length, with values of >0.8.

The hypocotyl is the only organ with an embryonic origin, and we detect that the hypocotyl length is the only trait that shows a negative correlation with the other measured characteristics. Moreover, the width of the hypocotyl shows a low and insignificant correlation with the remaining traits. Notably, there is a strong negative correlation between hypocotyl length and stem length (Fig. 9). This is evident during the developmental stages, because we observe that the length of the hypocotyl decreases over time, whereas the stem length increases (Fig. 5).

In Cactaceae, the SAM is among the largest described in plants. According to Mauseth (2004), there is a positive correlation between the diameter of the stem and the size of the SAM. Our observations revealed a positive correlation between dome width and stem width (0.72, s.d. ±0.49). Surprisingly, the highest correlation was found between dome width and stem length (1, s.d. ±0.00). These strong correlations are observed only in the latest four stages (5–8), as illustrated in the correlation graphs (see Supplementary Data Fig. S1). These data indicate that the SAM architecture can effectively correlate the width and length of the stem, similar to findings in other Cactaceae species (Mauseth, 2004). In other words, our data show that the ontogenetic allometric relationship between SAM and stem development recapitulates the trends observed at the cacti phylogenetic level.

## DISCUSSION

### *Mammillaria san-angelensis* has big SAMs that grow over time and are positively correlated with stem width and length

The largest apical meristems observed in angiosperms are found in the Cactaceae family (Boke, 1980; Mauseth, 2004), and the size of the SAM is positively correlated with the diameter of the stem (Mauseth, 2004). For instance, in large barrel cacti, such as *Echinocactus platyacanthus*, the SAM can reach ≤2.5 mm in diameter, with a stem diameter of 66.2 cm, compared with the *A. thaliana* SAM that reaches a maximum width of 60–70 μm (Medford *et al.*, 1992; Barton, 2010). Interestingly, even smaller cacti, such as members of the genera *Echinocereus*, *Coryphantha*, *Gymnocalycium* and *Mammillaria*, typically have SAMs with diameters ranging from 0.6 to 1.5 mm (Boke, 1951, 1955, 1958; Mauseth, 2004).

We discovered that at germination, the SAM dome width until stage 5 (30 dag) does not change significantly, reaching up to 136.964 (s.d. ±24.853) µm. However, at stage 6, it doubles in size, until reaching 771.71 (s.d. ±64.533) µm at 4 yag. These results agree with previous studies, in which some *Mammillaria* species have been characterized by their large SAMs in adult individuals; however, no chronological sequence of development had been described before in the seedling stage. For instance, *Mammillaria heyderi* has a stem diameter of 9–12 cm, and its SAM reaches a maximum diameter of ∼1200–1500 µm. At the time of its description in 1953, the data indicated that *M. heyderi* had the largest SAM documented for any flowering plant (Boke, 1953).

We found that the width, perimeter and length of the SAM were positively correlated with stem width. Importantly, for the first time in cacti, we found a strong correlation between the width of the SAM and stem length, which has not been described previously. This correlation has been described in chickpea, in which various morphometric traits of the SAM, such as the width, height and area, serve as key indicators for understanding the genetic basis of phenotypic variation observed in plant width and height (Narnoliya *et al.*, 2019). Furthermore, it has been described that in several herbaceous plants the stem width is correlated with the shape of the SAM, which is defined by the number of cells at the inner periphery and the number of cells across the thickness of the meristem (Schnablová *et al.*, 2017). In maize seedling SAM size, there is a correlation with agronomically important adult traits, such as flowering time, stem size and number of leaf nodes, besides the identification of candidate genes controlling these traits (Leiboff *et al.*, 2015).

It appears that larger SAMs are associated with stem succulence in cacti. In early-divergent cacti, particularly those from the Pereskioideae subfamily, the stems are not succulent. Among the earliest diverging species, *Pereskia aculeata* has the smallest shoot apex, measuring between 80 and 120 µm (Boke, 1954). This species also features the most slender stems and the fewest spines compared with other *Pereskia* species, such as *Pereskia grandifolia*, which is supposed to be more recently divergent and has a shoot apex size of 320 µm (Boke, 1954). In other words, larger SAMs seem to be derived characters in cacti.

### The shape of the SAM undergoes changes from the germination stage to its mature form, highlighting the dynamic process of plant development

After germination, some species may not exhibit a fully developed SAM, including complete zonation (Mauseth, 1978; Miravel-Gabriel *et al.*, 2024). In some cases, the SAM zonation is complete in the embryo before germination, but in others it may not be established fully until some time after the 30th plastochron (Mauseth, 1978). Specifically, in *Mammillaria brandegeei*, germination displays a concave meristem (Mauseth, 1978), which is in line with what we found in some individuals at stage 1 (1 dag), with some seedlings having a flat or slightly dome-shaped SAM. However, the complete chronology of SAM development in cacti remains unknown, because no studies have examined its formation during embryogenesis. In *A. thaliana*, the SAM develops during embryogenesis. In the eight-cell stage, the upper tier, consisting of four cells, serves as the founders of the shoot, including the SAM. In contrast, the lower tier is responsible for giving rise to the hypocotyl and the embryonic root (Boscá, 2011). In *M. san-angelensis*, the hypocotyl width shows the lowest correlations with the other analysed traits, and the hypocotyl length has negative correlations.

**Fig. 10.**
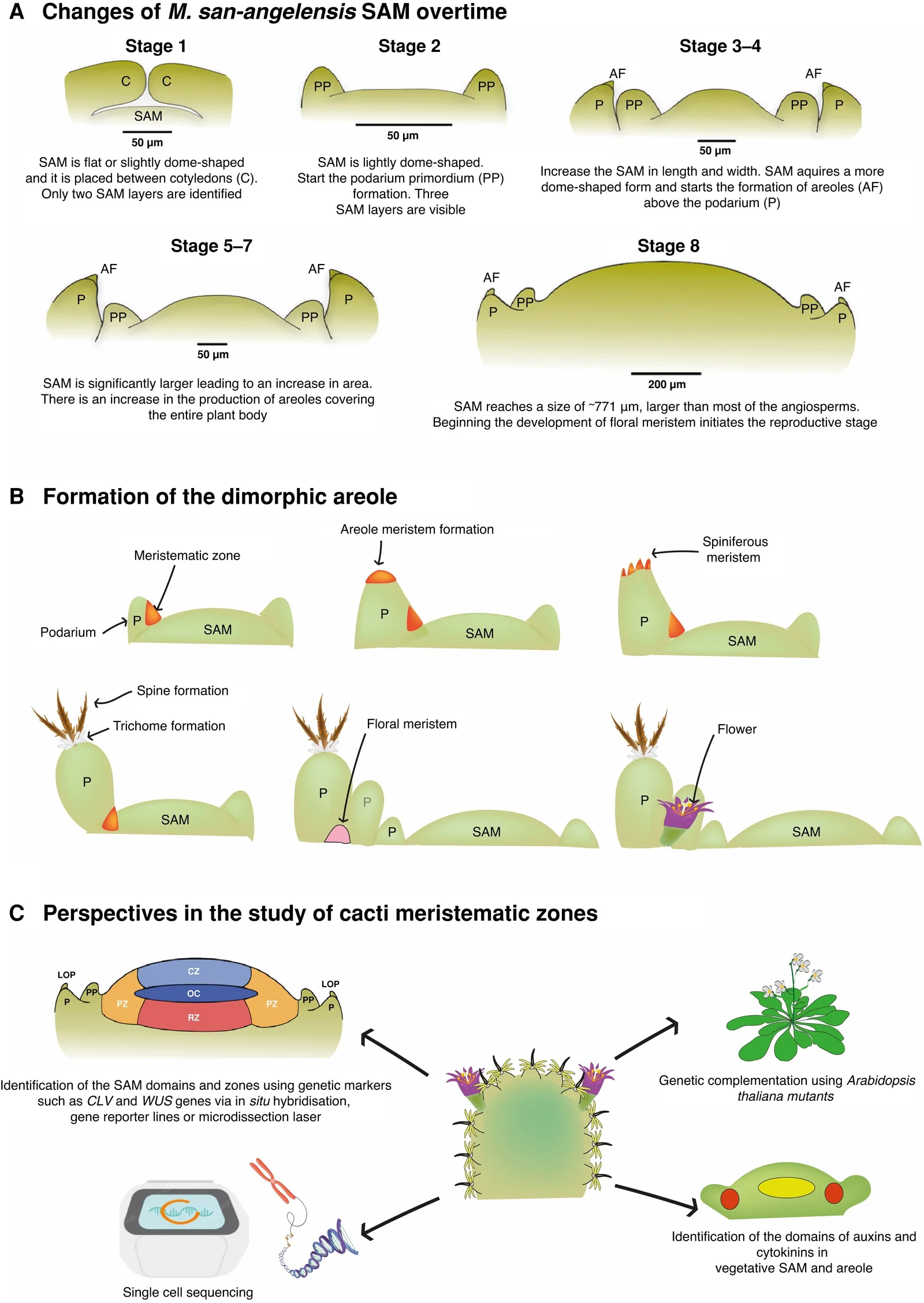
Models describing the shoot apical meristem (SAM) morphogenesis of *Mammillaria san-angelensi*s from 1 day after germination to 4-year-old plants, dimorphic areole formation, and future perspectives in the study of meristematic zones in Cactaceae. (A) The primary changes observed include the formation of a dome-shaped SAM, the transition from two to three layers, and the increase in SAM size. (B) The dimorphic areole begins to develop on the flanks of the SAM as a meristematic bulge, which then enlarges to form the podarium (P). At the summit of the podarium, a meristematic zone forms and develops into the areole. Initially, in the areole, the spiniferous meristem forms spines, followed by the formation of trichomes. Eventually, in the axil of the podarium, the flower meristem develops, leading to the formation of flowers. (C) Future studies on the meristematic zones in Cactaceae should focus on identifying genes that are important for SAM size, such as *WUS*, *CLV* and *STM*. Techniques such as reporter lines, *in situ* hybridization or laser microdissection can be used to establish the zones and domains of the SAM, as demonstrated in *Arabidopsis thaliana*. Additionally, performing single-cell RNA sequencing can help to map the transcriptome of each cell, in addition to identification of hormone localization, such as cytokinins and auxins in the SAM. Complementation analysis, by introducing orthologous genes from cacti into *A. thaliana* loss-of-function mutants with SAM defects could also be a valuable method to determine if the giant phenotype of the SAM can be recovered.

By the heart stage of *A. thaliana* embryogenesis, the SAM has developed into three layers of cells, which serve as the precursors to the L1, L2 and L3 layers; once the embryo is fully formed, the SAM takes on a slightly domed shape (Barton and Poethig, 1993). In *M. san-angelensis*, the SAM dome becomes visible at 3 dag, and by 15 dag the SAM has a well-defined dome with three layers that remain until stage 8 (4 yag). However, at germination (1 dag), only two layers are distinguishable. Modification of the SAM architecture over time also happens in *A. thaliana*. After germination, the 2-day-old *A. thaliana* seedling has a rectangular shape and is flat; at 5 days it becomes trapezoidal, and by 7 days old, the meristem takes on a complete dome shape (Medford *et al.*, 1992). In our model plant, we observe how the SAM changes from a flat, slightly dome-shaped structure to a more well-defined dome (Fig. 10). Additionally, the SAMs of other cactus seedlings enlarge significantly during vegetative development, showing notable differences in size in comparison to the model plants. Interestingly, we observed interspecific parallelism in SAM development during the developmental stages of *M. san-angelensis*, similar to that observed in other members of the Cactaceae family. As the stem increases, the SAM also increases, demonstrating the positive correlation previously described by Mauseth (2004) (Supplementary Data Fig. S1).

### The meristematic zone of the apical areole produces spines and trichomes but does not form a dome

It is well established that species within the *Mammillaria* genus exhibit dimorphic areoles: one is formed at the apex of the podarium, whereas the other arises from the axil. At the apex of the podarium, spines are produced; the first spines that appear are located abaxially to the areole meristem, eventually forming a ring of spines (Boke, 1953).

It has been suggested that dimorphic areoles in *Mammillaria* arise from the elongation and division of a single areole meristem representing a type of dichotomy (Buxbaum, 1951). Although the origin of an areole remains a mystery, the beginnings of dimorphic areoles are still entirely unknown. We observed that at germination, the SAM is placed between cotyledons, but eventually, it will form the podarium primordium as a bulge, which enlarges, and by day 7 (stage 3) the areoles are visible. However, we do not observe a meristematic dome at the top of the podarium where the areole is formed. The areole could be considered as an axillary meristem, whereas the podarium could be regarded as a leaf base. An interesting observation made ∼70 years ago described how the surface layer of cacti remains meristematic for a considerable distance from the SAM, which was called the peripheral primary meristem (PPM; Boke, 1944, 1951, 1952, 1953). The PPM will form the areole and the floral meristem in the axil of the podarium. Besides, it has been described that leaf primordia develop on both sides of the areole, arising before the formation of spine primordia (Boke, 1953). However, we did not detect any structures that could resemble a leaf. We believe that there is insufficient evidence to support the idea that the podarium is a leaf base and, at its summit, a leaf proper is formed. Thus, developmental markers (i.e. gene expression profiles or hormone activities) are necessary to determine their similarity to a leaf, such as auxin maximum that marks leaf initiation and *STM* downregulation (Bar and Ori, 2014; Wang *et al.*, 2021; Li *et al.*, 2024).

In angiosperms, specifically in *A. thaliana*, the SAM produces leaf primordia in the PZ that will develop into mature leaves. Axillary meristems then form within the axils of these growing leaves (Hirano and Tanaka, 2020). Axillary meristems do not develop during the seedling stage of *A. thaliana*; they form after Plastochron15–Plastochron21 have developed in the adult stage (Yang *et al.*, 2024). In cacti, however, a different developmental programme must occur. The areole, which is considered an axillary meristem, develops at 3 dag. Furthermore, an additional meristem is described in cacti: the spiniferous meristem that generates spines. The spiniferous meristem initiates near the adaxial side of the rudimentary leaf, where one series of spine primordia encircles the meristem (Boke, 1980). Because our anatomical sections are longitudinal, we were unable to observe all the spine primordia in the areole. Once again, we believe that genetic and molecular markers of development could help to clarify whether the spines are leaf derived.

### The formation of floral meristems occurs in the podarium base

In *M. san-angelensis*, flowers develop at the base of the podarium when the plant reaches ∼4 years of age. Floral primordia develop in the axil of certain podaria after the spines have formed (Boke, 1953). The flowering areole forms a single flower and can also produce trichomes, which have been suggested to be homologous to spines.

The literature outlines the chronological sequence of structure formation following the vegetative SAM formation, as follows: (1) the formation of the podarium primordia (considered as the base of the leaf); (2) the development of the leaf itself (named the leaf proper); (3) the emergence of spines and trichomes; and (4) the formation of flowers (Boke, 1944, 1953).

*Mammillaria san-angelensis* is not described as having cespitose growth; however, a remarkable feature is its ability to regenerate after the apex is decapitated. Regeneration occurs at the site of the cut, forming a main apex that eventually starts branching (Boke, 1980).

Finally, we emphasize that research on cacti should enter the era of molecular genetics and genomics more ambitiously. These approaches can uncover the fundamental mechanisms that underline meristems in cacti, the origins of gigantism and dimorphic areoles, and their ability to form different organs in a single region. As stated by Boke (1953), ‘these large apical meristems should someday provide excellent material for experimental morphologists’.

### Conclusion

This study is the first to describe the morphogenesis of the SAM, podarium and areoles in the Mexican cactus *M. san-angelensis*. We identified key developmental processes during the early stages of plantlet growth and their association with SAM growth. Our findings indicate that SAM size can explain the allometric growth of the stem width and length. SAM size has previously been correlated with stem size in adult plants across the cacti family (Mauseth, 2004).

In *M. san-angelensis*, during the initial developmental stages, the SAM transitions from a slightly flat to a meristematic dome, reaching a maximum average width of 771.71 (s.d. ±64.53) μm, along with the establishment of three layers. Statistically significant changes in all SAM traits were observed only at stages 6–8. A summary of the changes in SAM over time is illustrated in Fig. 10A. The initiation of dimorphic areoles begins at stage 2 (3 dag), starting as a meristematic bulge that elongates to form a meristem for formation of spines at the summit of the podarium. Additionally, the floral meristem is established by stage 8. All of the developmental stages for dimorphic areole formation are summarized in Fig. 10B.

Overall, we have established a developmental analysis for future genetic research aimed at characterizing the factors contributing to the increase in SAM size, in addition to the meristem variants of *M. san-angelensis*. We are currently working on generating transcriptomic and genomic information to consolidate *M. san-angelensis* as a model system; for that, these developmental stages that we have described are highly relevant.

Future research will focus on identifying genes that are significant for SAM size, such as *WUS*, *CLV* and *STM.* This will also involve recognizing zones and domains within the SAM using reporter lines, *in situ* hybridization, laser microdissection, single-cell RNA sequencing and examination of the roles of relevant hormones, such as cytokinins and auxins in the SAM (Fig. 10C). Furthermore, genetic complementation analysis, by introducing orthologous genes from cacti into *A. thaliana* loss-of-function mutants with SAM defects, could offer valuable insights into the functions of putative cactus genes. We believe that these studies will help to explain the origins of gigantism, succulence and the diversity of shapes and sizes within the Cactaceae family.

## Supplementary Material

mcag130_Supplementary_Data

## SUPPLEMENTARY DATA

Supplementary data are available at *Annals of Botany* online. Figure S1: the scatterplot displays data from the eight developmental stages, where the plantlet and SAM variables were log_1_  _+_  *_x_*-transformed. The mean of each variable at each stage was used to create the scatterplots for each unique pair of variables. Each scatterplot is color-coded by developmental stage and includes a fitted linear regression trend line. The coefficient of determination (R²) is annotated in the top corner of each plot. The strong correlations are observed mainly in the latest four stages (5th, 6th, 7th, and 8th). Table S1: measurements for the plant traits, hypocotyl, stem length and width. Table S2: measurements of SAM: width, length, and perimeter of dome and SAM area and perimeter.

## AI ASSISTANCE

Grammarly was used to check the grammar, spelling and punctuation of the text.
