# Supplementary material for: Shoot apical meristem morphoanatomy of the Mexican cactus Mammillaria san-angelensis and its significance for evolutionary developmental biology
Source: Ann Bot. 2026 Jun 1;138(4):1325–42. doi: 10.1093/aob/mcag130 (PMC13595257; doi:10.1093/aob/mcag130)
Supplement: Supplementary file 1 [file mcag130_supplementary_data.zip › Figure S1.docx]

**Figure S1**. The scatterplot displays data from the eight developmental stages, where the plantlet and SAM variables were log₁₊ₓ-transformed. The mean of each variable at each stage was used to create the scatterplots for each unique pair of variables. Each scatterplot is color-coded by developmental stage and includes a fitted linear regression trend line. The coefficient of determination (R²) is annotated in the top corner of each plot. The strong correlations are observed mainly in the latest four stages (5^th^, 6^th^, 7^th^, and 8^th^).
